# A Retrospective Analysis about Frequency of Monitoring in Italian Chronic Myeloid Leukemia Patients after Discontinuation

**DOI:** 10.3390/jcm9113692

**Published:** 2020-11-17

**Authors:** Matteo Dragani, Giovanna Rege Cambrin, Paola Berchialla, Irene Dogliotti, Gianantonio Rosti, Fausto Castagnetti, Isabella Capodanno, Bruno Martino, Marco Cerrano, Dario Ferrero, Carlo Gambacorti-Passerini, Monica Crugnola, Chiara Elena, Massimo Breccia, Alessandra Iurlo, Daniele Cattaneo, Sara Galimberti, Antonella Gozzini, Monica Bocchia, Francesca Lunghi, Michele Cedrone, Nicola Sgherza, Luigia Luciano, Sabina Russo, Marco Santoro, Valentina Giai, Giovanni Caocci, Luciano Levato, Elisabetta Abruzzese, Federica Sora, Giuseppe Saglio, Carmen Fava

**Affiliations:** 1 Department of Clinical and Biological Sciences, University of Turin, 10043 Orbassano, Italy; giovanna.rege@libero.it (G.R.C.); paola.berchialla@unito.it (P.B.); giuseppe.saglio@unito.it (G.S.); carmen.fava@unito.it (C.F.); 2 Hematology Unit, Department of Biotechnology and Health Sciences, University of Turin, 10126 Turin, Italy; irenedogl@hotmail.it (I.D.); cerranomarco@gmail.com (M.C.); dario.ferrero@unito.it (D.F.); 3 Institute of Hematology “L. e A. Seràgnoli”, University of Bologna, 40138 Bologna, Italy; gianantonio.rosti@unibo.it (G.R.); fausto.castagnetti@unibo.it (F.C.); 4 Hematology Unit, Azienda Unità Sanitaria Locale-IRCCS, 42123 Reggio Emilia, Italy; isabella.capodanno@ausl.re.it; 5 Hematology Unit, Grande Ospedale Metropolitano “Bianchi-Melacrino-Morelli”, 89124 Reggio Calabria, Italy; brunmartin54@gmail.com; 6 Hematology Division, San Gerardo Hospital, 20900 Monza, Italy; carlo.gambacorti@unimib.it; 7 Division of Hematology, Azienda Ospedaliero-Universitaria di Parma, 43126 Parma, Italy; mcrugnola@ao.pr.it; 8 Department of Hematology Oncology, Foundation IRCCS Policlinico San Matteo, University of Pavia, 27100 Pavia, Italy; chiara.elena1@gmail.com; 9 Department of Translational and Precision Medicine, Sapienza University, 00161 Rome, Italy; breccia@bce.uniroma1.it; 10 Hematology Division, Foundation IRCCS Ca’ Granda Ospedale Maggiore Policlinico, 20122 Milan, Italy; alessandra.iurlo@policlinico.mi.it (A.I.); daniele.cattaneo@policlinico.mi.it (D.C.); 11 Hematology Department, University of Pisa, 56126 Pisa, Italy; sara.galimberti@med.unipi.it; 12 Hematology Division, Policlinico Careggi di Firenze, 50139 Firenze, Italy; antonella.gozzini@unifi.it; 13 Hematology Unit, University of Siena, Azienda Ospedaliera Universitaria Senese, 53100 Siena, Italy; monica.bocchia@unisi.it; 14 Hematology and Bone Marrow Transplant Unit, San Raffaele Scientific Institute IRCCS, 20132 Milano, Italy; lunghi.francesca@hsr.it; 15 Hematology Division, Az. Ospedaliera San Giovanni Addolorata, 00184 Rome, Italy; michele.cedrone@gmail.com; 16 Division of Hematology, IRCCS Ospedale Casa Sollievo Sofferenza, 71043 San Giovanni Rotondo, Italy; nicolasgherza@libero.it; 17 Hematology Unit, “Federico II” Hospital, University of Naples, 80131 Napoli, Italy; lulucian@unina.it; 18 Department of Internal Medicine, AOU Policlinico di Messina, 98124 Messina, Italy; sabinarusso20@gmail.com; 19 Hematology Unit, University of Palermo, 90127 Palermo, Italy; santoro.dott@gmail.com; 20 Hematology Unit, Antonio e Biagio e Cesare Arrigo Hospital, 15121 Alessandria, Italy; valentina.giai@unito.it; 21 Hematology Unit, Department of Medical Sciences, University of Cagliari, 09121 Cagliari, Italy; giovanni.caocci@unica.it; 22 Department Hematology-Oncology, Azienda Ospedaliera Pugliese-Ciaccio, 88100 Catanzaro, Italy; leluc13@alice.it; 23 Hematology Unit, S. Eugenio Hospital, Tor Vergata University, 00144 Rome, Italy; elisabetta.abruzzese@uniroma2.it; 24 Hematology Unit, Fondazione Policlinico Universitario Gemelli IRCCS, 00168 Rome, Italy; federica.sora@unicatt.it

**Keywords:** chronic myeloid leukemia, treatment-free remission, molecular monitoring

## Abstract

Successful discontinuation of tyrosine kinase inhibitors has been achieved in patients with chronic-phase chronic myeloid leukemia (CML). Careful molecular monitoring after discontinuation warrants safe and prompt resumption of therapy. We retrospectively evaluated how molecular monitoring has been conducted in Italy in a cohort of patients who discontinued tyrosine kinase inhibitor (TKI) treatment per clinical practice. The outcome of these patients has recently been reported—281 chronic-phase CML patients were included in this subanalysis. Median follow-up since discontinuation was 2 years. Overall, 2203 analyses were performed, 17.9% in the first three months and 38.4% in the first six months. Eighty-six patients lost major molecular response (MMR) in a mean time of 5.7 months—65 pts (75.6%) during the first six months. We evaluated the number of patients who would experience a delay in diagnosis of MMR loss if a three-month monitoring schedule was adopted. In the first 6 months, 19 pts (29.2%) would have a one-month delay, 26 (40%) a 2-month delay. Very few patients would experience a delay in the following months. A less intense frequency of monitoring, particularly after the first 6 months off treatment, would not have affected the success of treatment-free remission (TFR) nor put patients at risk of progression.

## 1. Background

Since the first proof of concept trial for stopping tyrosine kinase inhibitors (TKIs), the STIM1 study, successful treatment-free remission (TFR) has been obtained in many patients with chronic-phase chronic myeloid leukemia (CP-CML), both after imatinib and after second generation TKIs (2GTKIs) [1,2,3,4,5,6,7].

Criteria for treatment discontinuation and for treatment resumption varied among the published studies and reported TFR rates ranged from 38% to 70% as well. However, there was high consistency across studies in terms of safety; extremely low rates of progression to accelerate/blast phase were observed, an encouraging aspect for the feasibility of TKI discontinuation also outside clinical trials. Moreover, more than 90% of patients who need to resume TKI therapy due to molecular recurrence are able to regain their initial deep molecular response state [8,9].

Stopping TKI treatment in selected patients is therefore now considered a safe procedure, at least for centers that have access to robust sensitive molecular monitoring, with a rapid turnaround time for results [8]. Remarkably, up to 80% of molecular relapses arise within the first 6 to 12 months from treatment discontinuation [10,11]; a particularly stringent monitoring is advised in the initial phases of TKI discontinuation to guarantee a prompt resumption of therapy according to retreatment threshold criteria. In fact, monthly quantitative PCR (qPCR) monitoring during the first half-year has been adopted by all prospective protocols to address safety concerns, and it has been used to establish the median BCR-ABL1 doubling time, equal to 9 days (6.9–25.5), in case of imatinib discontinuation after sustained complete molecular remission (CMR) [1,4,8,12,13].

On the other hand, a stringent monitoring schedule even outside clinical trials necessarily requires patients to access the referral hematology center frequently, more often than during active treatment, an aspect that needs to be carefully discussed with patients attempting to achieve TFR.

Moreover, while standard of care (i.e., every three months) CML molecular monitoring, compared to no monitoring, has been proven to be cost-effective in terms of savings derived from prevention of disease progression, intensified monitoring during TFR is associated with higher—at least immediate—costs [14]. Taken together, logistic and cost issues may prevent some patients from attempting to achieve TFR, in particular in countries with limited funding for molecular evaluation.

Two studies recently investigated if performing molecular analysis with a different and less “cautious” timeframe would yield a comparable efficacy with logistical issues and cost reduction [12,15]. The first study adopted a monthly approach for the first three months, followed by qPCR analyses every three months for 1 year and every six months thereafter; in this case, limited by a small sample size (*n* = 24), no progression was observed and the TFR rate was 66% at 2 years of follow-up.

In the study by Shanmuganathan et al., different monitoring algorithms were tested and compared to current National Comprehensive Cancer Network (NCCN) guidelines to estimate the possible delay in relapse detection and TKI therapy resumption; the eventual loss of cytogenetic response with each model was predicted by calculating the BCR-ABL1 doubling time in an actual cohort of TFR patients. Interestingly, less frequent monitoring, i.e., every two months in the first six months and every three months between six and twelve months, resulted in superior cost-effectiveness [15].

Here, we retrospectively evaluated how molecular monitoring has been conducted in Italy on a multicentric cohort of patients not included in any prospective trial; of note, thresholds for treatment discontinuation and resumption were not pre-defined across centers, and patients were considered candidates for discontinuation in case of a sustained deep molecular response (DMR), defined as MR4 (BCR-ABL1 ratio ≤ 0.01% with at least 10,000 ABL1 copies), or MR4.5 (BCR-ABL1 ratio ≤ 0.0032% with at least 32,000 ABL1 copies), or MR5 (BCR-ABL1 ratio ≤ 0.001% with at least 100,000 ABL1 copies), confirmed at least three times [3].

## 2. Materials and Methods

The characteristics and outcome of Italian patients with CP-CML who discontinued TKIs have recently been reported [3]. Briefly, all patients who had discontinued TKI treatment per clinical practice while in deep molecular remission were eligible for the study, provided a minimum follow-up after discontinuation of 2 years was available. All the Hematological Centers that belong to the Italian Group for the Hematologic Diseases of the Adults (GIMEMA) were invited to participate. The primary endpoint was the rate of TFR at one-year from TKI treatment discontinuation. Secondary safety endpoints included, among others, outcome after treatment resumption and disease progression.

For the purpose of the present study, all the 32 participating centers were required to provide dates and molecular results available for each enrolled patient in the first 24 months after TKI cessation.

Molecular responses were assessed by standard quantitative polymerase chain reaction (qPCR) [14,16]; molecular analyses were performed by the GIMEMA Laboratories Network (LabNet) for CML and expressed using the International Scale. The LabNet network assures reliability of results because it includes only standardized laboratories which periodically undergo quality control rounds; the latter are essential to re-determine the laboratory-specific conversion factor each year, which is multiplied by the percentage value of BCR-ABL1/ABL1 to finally obtain the measurement of BCR-ABL^IS^.

Major Molecular Response (MMR) was defined as a BCR-ABL1 ratio ≤ 0.1 with at least 10,000 ABL1 copies; MMR loss was defined as 1 single BCR-ABL1 quantification above 0.1%.

Descriptive statistical analysis was carried out. The average time to loss of MMR, the frequency of the visits (monitoring) and the occurrence of loss of MMR within the first 6 months, between 6 and 12 months and 13 and 24 months were computed. When appropriate, a Kruskal–Wallis test was used to test for differences between more than two groups.

The level of statistical significance was set at 0.05.

Statistical analyses were carried out using R version 4.0.

## 3. Results

**Figure 1:**
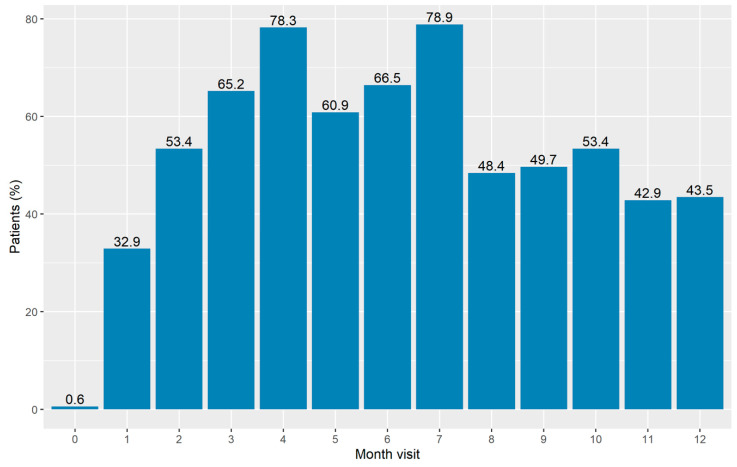
Percentage of patients who had a minimal residual disease assessment during the first 12 months in our retrospective cohort.

Two-hundred and eighty-one chronic-phase CML patients were included in this subanalysis. Median age at TKI discontinuation was 60.1 (IQR—interquartile range: 48.2–69.8) years and median follow-up since TFR was 2.3 years (IQR 2.0–3.1). For this cohort the median duration of sustained DMRs before treatment interruption was 3.3 years (IQR 2.3–5.9). In this timeframe every patient had a mean of 7.8 (±4.7) appointments for molecular evaluation. Overall, 2203 analyses were performed, of which 17.9% happened in the first three months and 38.4% in the first six months. During the first three months of TKI discontinuation, 59 patients (21%) did not have any molecular assessment; 1 qPCR was performed for 93 patients (33.1%), 2 qPCRs for 88 patients (31.3%), 3 qPCRs for 39 patients (13.9%) and 4 qPCRs for two patients (0.7%). For the first six months after TKI stop, 9 patients (3.2%) did not undergo any BCR-ABL1 evaluation; 55 patients (19.6%) only underwent one analysis, 72 patients (25.6%) underwent two analyses, 38 patients (13.5%) underwent three analyses, 30 patients (10.7%) were evaluated four times, 51 patients (18.1%) five times, 25 patients (8.9%) six times and only 1 patient (0.4%) seven times. The majority of visits fell between the third and the seventh month after TKI interruption (Figure 1) with 105 patients (65.2%) evaluated at month 3, 126 patients (78.3%) at month 4, 98 patients (60.9%) at month 5, 107 patients (66.5%) at month 6 and 127 patients (78.9%) at month 7.

In the first six months the visits occurred with a mean interval of 1.43 (±0.95) months; between months 7 and 12 molecular evaluations were performed every 2.02 (±1.34) months; during the second year of discontinuation (months 13–24) every 3.04 (±1.99) months (*p* < 0.001, Kruskal–Wallis test). Eighty-six patients lost major molecular response (MMR) in a mean time of 5.7 (±4.3) months. As expected, 65 patients (23.13% of the initial 281 patients) lost MMR during the first six months whereas 21 patients (24.4%) relapsed later on: 3 patients (3.5%) relapsed within the first month, 7 pts (8.1%) in the second, 14 pts (16.3%) in the third, 23 (26.7%) in the fourth, 12 (14%) in the fifth and 6 patients (7%) in the sixth month. Thirteen patients lost MMR during the second semester (4.62% of the initial 281 patients): 7 patients in the seventh month, 3 patients in the eightieth, 1 patient in the ninth, 1 patient in the eleventh and 1 patient in the twelfth. Only 8 (2.84%) patients lost MMR after 12 months of follow-up in TFR (Table 1).

Median BCR-ABL1 value at MMR loss for the 65 patients who relapsed in the first six months was 0.40 (IQR 0.181–0.24); it was 0.32 (IQR 0.151–0.36) for the 13 subjects who lost MMR in the second semester and 0.26 (IQR 0.14, 0.78) for the 8 patients who lost MMR after the first year of discontinuation. No significant differences were found in BCR-ABL1 values at MMR loss between the three mentioned groups (*p* = 0.64, Kruskal–Wallis test). 

We sought to determine if the depth of molecular remission at treatment discontinuation influenced the velocity of MMR loss; data to perform this analysis were available for 174 patients—we did not find any difference in time length between TKI interruption and MMR loss amongst patients in MMR, MR4, MR4.5 and MR5 before treatment stop (Table 2).

All patients regained at least MMR after TKI resumption, and no progression occurred. Among the 86 patients who restarted therapy, we had information about the type of treatment which was resumed for 85 of them: 16 patients (18.8%) experienced a switch to a different TKI compared with the one that was stopped at the moment they attempted TFR.

**Figure 2:**
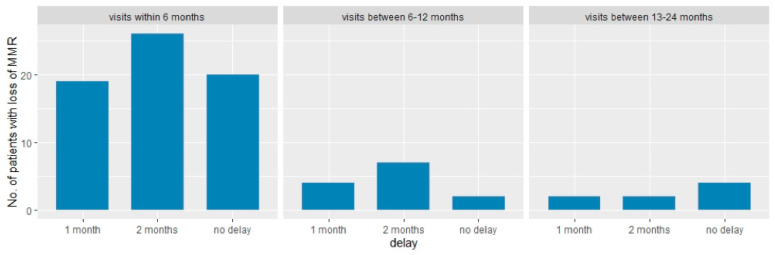
Delay of MMR (major molecular response) loss detection by timeframe if 3-month monitoring schedule is applied in our cohort.

Finally, we evaluated the number of patients who would experience a delay in the diagnosis of MMR loss (and consequently a delay in treatment resumption) if a three-month monitoring schedule was adopted. In the first 6 months, 19 patients (29.2%) would have a one-month delay, 26 (40%) a 2-month delay; 20 patients (30.8%) would experience no delay. Very few patients would experience a delay in the following months (Figure 2).

## 4. Discussion

Here we present the results about the frequency of molecular monitoring in our retrospective cohort of patients who interrupted their treatment per clinical practice in Italy. Two-hundred and eighty-one patients who attempted TFR were monitored at different time points which were not established a priori. Despite 85% of the patients in the first 3 months and 91% within the first 6 months receiving lesser qPCR monitoring than recommended by guidelines, they experienced a satisfactory TFR rate without any progression to advanced phases and with prompt re-gain of MMR after TKI resumption. Depth of molecular remission at TKI cessation did not influence the velocity of MMR loss in our cohort, failing to identify a molecular category at discontinuation that may have a diverse kinetic of relapse and, consequently, the need to be monitored differently. Analyzing median BCR-ABL1 values at MMR loss in patients who relapsed, we found that the monitoring schedule applied here was able to catch patients facing MMR loss while remaining in complete cytogenetic response (associated with BCR-ABL1 < 1%) especially in the second year of discontinuation, where the median BCR-ABL1 value and its IQRs were 0.26 and 0.14 –0.78, respectively. Looking instead at the IQRs of MMR values in patients who relapsed within the first year of discontinuation (IQR 0.18–1.24 in the first 6 months and IQR 0.15–1.36 during the second semester), it appears that 1 patient out of 4 had a transcript that crossed the threshold of 1%, thus advocating a more strict monitoring schedule during the first year of TKI interruption. Additionally, we showed that by applying a trimestral monitoring schedule, the 29.2% and 40% of patients who lost MMR would have experienced a 1-month and 2-month delay, respectively, in its detection, and consequentially in TKI retreatment.

The safety of TFR relies on the management of patients off therapy, especially during the first 6 months, when molecular relapses occur more often and require a more stringent follow-up for early detection of MMR loss, typically characterized by an exponential rise in the BCR-ABL transcript. The timing of the analysis as reported by guidelines such as NCCN, European Society for Medical Oncology (ESMO) and European Leukemia Net (ELN) is based principally on the practices adopted by the first groups who attempted discontinuation such as EURO-SKI and STIM (Table 3) [1,4,8,17,18].

In light of the recent advances in TKI drug development as well as in molecular techniques, some additional variables need to be taken into consideration to determine the optimal timing of monitoring during TFR.

First, the availability of new methods for the detection of BCR-ABL minimal residual disease: the digital PCR (dPCR) is a new molecular tool which provides a very sensitive detection of low level of disease and whose results in terms of minimal residual quantification are more reproducible between laboratories, given the independence of this technique from the necessity of using calibration curves as happens in qPCR. It presents as a more predictive technique for TFR and two trials found that patients with a negative minimal residual disease (MRD) at dPCR have a better probability of sustained MMR than patients who are positive in the dPCR. Digital PCR positive patients have a median time to relapse of 5 months vs. “not reached” in the cohort of patients who are dPCR negative [19,20,21]. Based on these results it can be speculated that a less frequent monitoring can be considered for patients whose BCR-ABL negativity has been attested by dPCR instead of (or in parallel with) qPCR. However, the predictive role of dPCR deserves to be further explored so as not to risk being imprudent.

Secondly, in recent years the approach to TKI discontinuation has changed, with experiences about de-escalation of therapy before definitive treatment interruption [22,23]. This novel strategy may imply a better identification of patients who were destined to relapse and to resume therapy, leaving only patients who maintain molecular stability during the de-escalation phase to proceed with treatment-free remission; thus, the necessity of a strict monthly monitoring during the first months of no treatment would be reduced.

Studies such as Nilo-RED and DESTINY have allowed patients with MMR, not reaching MR4, to participate in TKI discontinuation. A quite successful rate of TFR has been observed in this subset of patients; however, translated in the real-life practice, such inclusive criteria would lead to maintaining a more frequent schedule of BCR-ABL testing, due to the higher level of disease at discontinuation.

On the other side, whereas a different molecular monitoring program would be appealing for patients and to reduce laboratory and economic burden, late relapses and the unpredictability of a sudden blast crisis should be taken into account in modelling future proposals: recently, 8 patients out of 114 (14%) experiencing a late molecular relapse were reported, of which four relapsed after the fifth year [24]; one patient who enrolled in the 2GTKI stop study was reported to progress onto myeloid blast crisis at sixth month of TKI reintroduction after losing MMR in an attempt of first line Nilotinib discontinuation, an event already reported in one patient of the Korean Imatinib Discontinuation (KID) study after Imatinib cessation, despite meeting all the criteria to attempt TFR [25,26]. Until prospective studies come out, we encourage following international recommendations of a once-a-month monitoring for the first six months, every six weeks during the second semester and every 3 months thereafter; nevertheless, our results suggest that a more relaxed timeframe of MRD assessment without risks need to be further explored in specific settings identified with the help of improved molecular techniques and prognostic scores.

## 5. Conclusions

Prospective studies are needed if a change of monitoring frequency is considered, reflecting the different selection criteria for TFR, the possibility to use predictive scores for successful TFR [27] and the novelties in MRD detection.

## Figures and Tables

**Table 1:** A panoramic of major molecular response (MMR) loss month by month. Percentages refer to the total number of relapses.

Months after Discontinuation	Patients with MMR Loss (%)
1	3 (3.5)
2	7 (8.1)
3	14 (16.3)
4	23 (26.7)
5	12 (14.0)
6	6 (7.0)
7	7 (8.1)
8	3 (3.5)
9	1 (1.2)
11	1 (1.2)
12	1 (1.2)
13+	8 (9.3)

**Table 2:** Depth of molecular remission at discontinuation and time to MMR loss.

	MR3	MR4	MR4.5	MR5	*p*
N° of patients	7	80	63	24	0.849 ^1^
Months between discontinuation and MMR loss (median (IQR))	3.14 [1.93, 6.83]	4.26 [2.94, 5.26]	3.44 [3.09, 3.85]	3.21 [3.17, 3.21]	

^1^ Kruskal–Wallis test.

**Table 3:** Timing of molecular monitoring in prospective trials and current guidelines.

Study	Timing of Monitoring	Study	Timing of Monitoring
STIM A-STIM	Once monthly for 12 months Once every 2 months for 12 months Once every 3 months thereafter	STOP2GTKI	Once monthly for 12 months Once every 2–3 months for 12 months Once every 36 months for up to 6 years
EURO-SKI	Once monthly for 6 months Once every 6 weeks for 6 months Once every 3 months thereafter	KID	Every month for 6 months Once every 2 months for 12 months Once every three months thereafter
DADI	Once monthly for 12 months Once every 3 months for 12 months Once every 6 months thereafter	LAST	Once monthly for 6 months Once every 2 months in months 7–24 Once every three months thereafter
JALGS- STIM213	Once monthly for 6 months Once every 2 months for 6 months Once every 3 months thereafter	ESMO	Once monthly for 6 months Every 6 weeks for 6 months Once every 3 months thereafter
ENEST freedom	Once monthly for 12 months Once every 6 weeks for 12 months Once every 3 months thereafter	NCCN	Once monthly for 12 months Every 6 weeks for 12 months Once every 3 months thereafter
TWISTER	Once monthly for 12 months Once every 2 months for 12 months Once every 3 months thereafter	ELN 2020	Once monthly for 6 months Once every 2 months for months 6–12 Once every three months thereafter
